# Development of a platform-specific objective assessment tool for the accreditation of robotic setup and docking

**DOI:** 10.1007/s00464-025-12311-1

**Published:** 2025-11-07

**Authors:** T. Shakir, G. Lingam, M. Boal, M. Chand, N. Francis

**Affiliations:** 1 University College London, 43-45 Foley St, London, UK; 2 St Marks Hospital and Academic Institute, London, UK; 3 The Griffin Institute, Harrow, UK

**Keywords:** Robotic surgery, Standardisation, Training, Assessment, Accreditation

## Abstract

**Background:**

Training in robotic surgery entails a stepwise progression, starting with technology training. This aspect, which includes setup and docking, is an intricate process, which can be performed by the wider surgical team, including trainees and surgical care practitioners. Despite this, no uniform accreditation framework exists for assessing competence in robotic system setup, with our aim to establish a robust mechanism for this.

**Methods:**

An international Delphi consensus was conducted to develop a stepwise accreditation protocol for robotic surgical setup. A granular checklist was developed by a steering committee, divided into five key phases of setup (system knowledge, port placement, docking, instrument exchange, and undocking) on the da Vinci Xi platform. Opinions on methods of accreditation, competency cut-offs, and assessors were also surveyed.

**Results:**

54 experts from 13 countries participated (73% consultant surgeons, 19% surgical care practitioners, 8% fellows). The majority had extensive robotic experience (61.9% involved in > 500 cases). Key findings included knowledge of port placement, the need for reverse communication with instrument changes, and standardised emergency undocking workflows. 85% of respondents supported certification for bedside assistants, preferably assessed by experienced robotic surgeons or assistants (82%). Based upon the protocol, competency was deemed to be demonstrated by a score of 80% or 90% by 81% of respondents. Statements were formulated into a freely usable online form.

**Conclusion:**

This Delphi consensus formulated a standardised accreditation curriculum for robotic system setup. The protocol provides a tool which can guide training and assessment of competency in robotic docking and setup, addressing a gap in robotic surgery training. Adoption of this framework could benefit training for surgical teams and improve safety. Future validation will be necessary to correlate use with improved performance.

Robotic surgery has seen rapid growth over the past two decades. Globally, over 2.25 million robotic procedures were performed in 2023, with cumulative totals exceeding 14 million [1]. In the same year, 1370 new surgical robots were installed worldwide. Whilst this expansion enables more advanced minimally invasive procedures, it also demands comprehensive training. This is not only for surgeons but also for the entire surgical team. Surgeons cannot progress with their training unless they have progressed through the technological learning phase [2, 3]. This includes learning how to manipulate the surgeon console, in addition to the patient-facing cart and docking process. With the expanding role of surgical care practitioners [4], robotic bedside assisting is often performed by these trained individuals who can assist across multiple specialties. There are therefore a number of individuals who require suitable training with respect to setup. Appropriate education is therefore paramount.

Training in robotic system setup is currently heterogeneous. Electronic learning modules and industry-led courses predominate, in addition to various surgeon-led courses. In the UK, multiple courses are available for basic robotic skills; however, these have relatively limited focus on docking and system setup [5]. These can be provided by Royal College of Surgeons accredited centers, surgical associations, or even hospitals. However, each course employs its own methods, incorporating personal perspectives, which can lead to inconsistency and potential inefficiencies or errors. The complexity of robotic platforms has multiple components requiring precise positioning and calibration. This makes standardisation of the setup component crucial for safety and reproducibility.

A related challenge is the lack of assessment for robotic bedside competence [6]. Despite the availability of robotic surgery training programmes, there is currently no uniform accreditation or certification process to evaluate an individual’s ability to set up and dock the robot [6]. Subjective processes make it difficult to ensure consistent standards, potentially affecting the quality of robotic training across different centres. Consensus documents have called for the need to combat fragmented training methods and to establish a uniform standard for training and assessment [7, 8].

To address this, we conducted an international Delphi consensus with the aim of defining a standardised protocol for robotic system setup. Using the da Vinci Xi platform, due to its global prevalence, we aimed to derive expert-agreed steps and criteria that could form the basis of a reproducible training and assessment framework. This manuscript focuses on the practical implementation of the resulting protocol, potentially providing a stepwise guide for accrediting bedside assistants in robotic surgery.

## Methods

A Delphi consensus regarding gold standard setup steps was conducted between June and September 2024. This was for a specific platform, the da Vinci Xi. This was chosen due to its global market lead [9]. A steering committee was formed of five experts (two consultant robotic surgeons, two experienced robotic surgical care practitioners, and one industry representative). A list of statements pertaining to setup and docking based on experience, literature, and training resources was developed. Relevant industry documentation and a robotic skills course manual [5] were also reviewed to ensure comprehensiveness. The initial list of proposed items was organised into five broad phases of robotic setup:

1. System knowledge
2. Port placement
3. Driving in & docking
4. Instruments and changes
5. Undocking & driving out

Each of these five aforementioned phases incorporated generic setup tasks at a granular level. 88 initial statements were formulated, with an additional 3 statements generated through participant feedback. This was distributed to robotic subcommittee members of international organisations including SAGES and EAES and speciality-specific committees. A standard 3-round Delphi process was followed, with consensus defined as being reached if responses were over an 80% threshold. In total, 85 statements drew consensus. These formulated the basis of expert-derived gold standard steps in setup, docking, and undocking of the robotic platform.

Experts were also surveyed during round 2 of the consensus regarding competence assessments. These questions were derived from the same steering committee and utilised the same online form for responses. Questions centred on their opinions for methods of accreditation, competency cut-offs, and who should be assessors (Table 1). These helped to guide the direction of the accreditation protocol.

**Table 1:** Robotic setup and docking accreditation survey questions

Who do you think this is a necessary competence for? (Check all that apply)
Do you think bedside assistants should be certified (with a form of assessment) for docking and setup?
Who do you think should certify?
How many cases would you define an expert in setup and docking?
Do you think a standardised process would be best *taught* by:
Do you think a standardised process would be best *assessed* by:
Do you think certification could be automated? (For example wearing a headset device with a training and assessment programme)
Based on this checklist, what score would you deem competent?

The statements were then formulated into an accessible format to guide curriculum and assessment. Descriptions were added to phases in order to allow flow in an examination format. The transcription of these onto an online form with yes/no answers for each statement and the associated scoring calculation allowed for a usable interface (Appendix 1).

## Results

### Survey results

54 participants responded from a total of 11 countries (Table 2). The majority were based in the UK (55.6%, n = 30), followed by Italy (9.3%, n = 5), Belgium (7.4%, n = 4), Germany and India (each 5.6%, n = 3), and the USA (3.7%, n = 2). Regarding roles, consultant/attendings comprised the largest group (74.1%, n = 40), with surgical care practitioners (14.8%, n = 8) and fellows (11.1%, n = 6) accounting for the remainder. Most reported high surgical experience, performing over 500 procedures (72.2%, n = 39), whilst fewer performed between 200 and 500 (16.7%, n = 9). The anatomical locations for these procedures were predominantly in the lower abdomen (57.4%, n = 31), with pelvic (27.8%, n = 15) and upper abdominal procedures (14.8%, n = 8) being less common.

**Table 2:** Demographics of survey respondents (*n* = 54)

		**n (%)**
**Country**	UK	30 (55.6%)
	Italy	5 (9.3%)
	Belgium	4 (7.4%)
	Germany	3 (5.6%)
	India	3 (5.6%)
	USA	2 (3.7%)
	Netherlands	2 (3.7%)
	Singapore	2 (3.7%)
	France	1 (1.9%)
	Greece	1 (1.9%)
	Hong Kong	1 (1.9%)
**Professional Role**	Consultant	40 (74.1%)
	First assistant	8 (14.8%)
	Fellow	6 (11.1%)
**Procedure Volume**	> 500	39 (72.2%)
	200–500	9 (16.7%)
	100–200	6 (11.1%)
**Procedure Location**	Lower abdomen	31 (57.4%)
	Pelvic	15 (27.8%)
	Upper abdomen	8 (14.8%)

Respondents universally identified robotic bedside assistants (54, 100.0%) as requiring this competence, closely followed by surgeons (49, 90.7%) and surgical trainees (45, 83.3%). A strong consensus (46 respondents, 85.2%) favoured mandatory certification for robotic bedside assistants, compared to 8 respondents (14.8%) who were opposed. Regarding who should certify, respondents predominantly supported established robotic surgeons (42, 77.8%), followed by established robotic assistants (31, 57.4%). An expert in robotic setup and docking was proposed as completion of 50–100 cases (37, 68.5%), with fewer advocating higher thresholds: 100–200 cases (12, 22.2%), 200–500 cases (3, 5.6%), and over 500 cases (2, 3.7%). Training preferences favoured in-person methodologies, with most respondents recommending training delivered by trained healthcare professionals (48, 88.9%) or industry representatives (36, 66.7%). Similarly, the preferred method for assessment was in-person evaluation by trained healthcare professionals (47, 87.0%). For checklist-based assessments, most participants selected an 80% threshold (27, 50.0%) as indicative of competence, followed by 90% (17, 31.5%), with no respondents opting for a threshold of 60%.

### Consensus outcomes

**Fig. 1:**
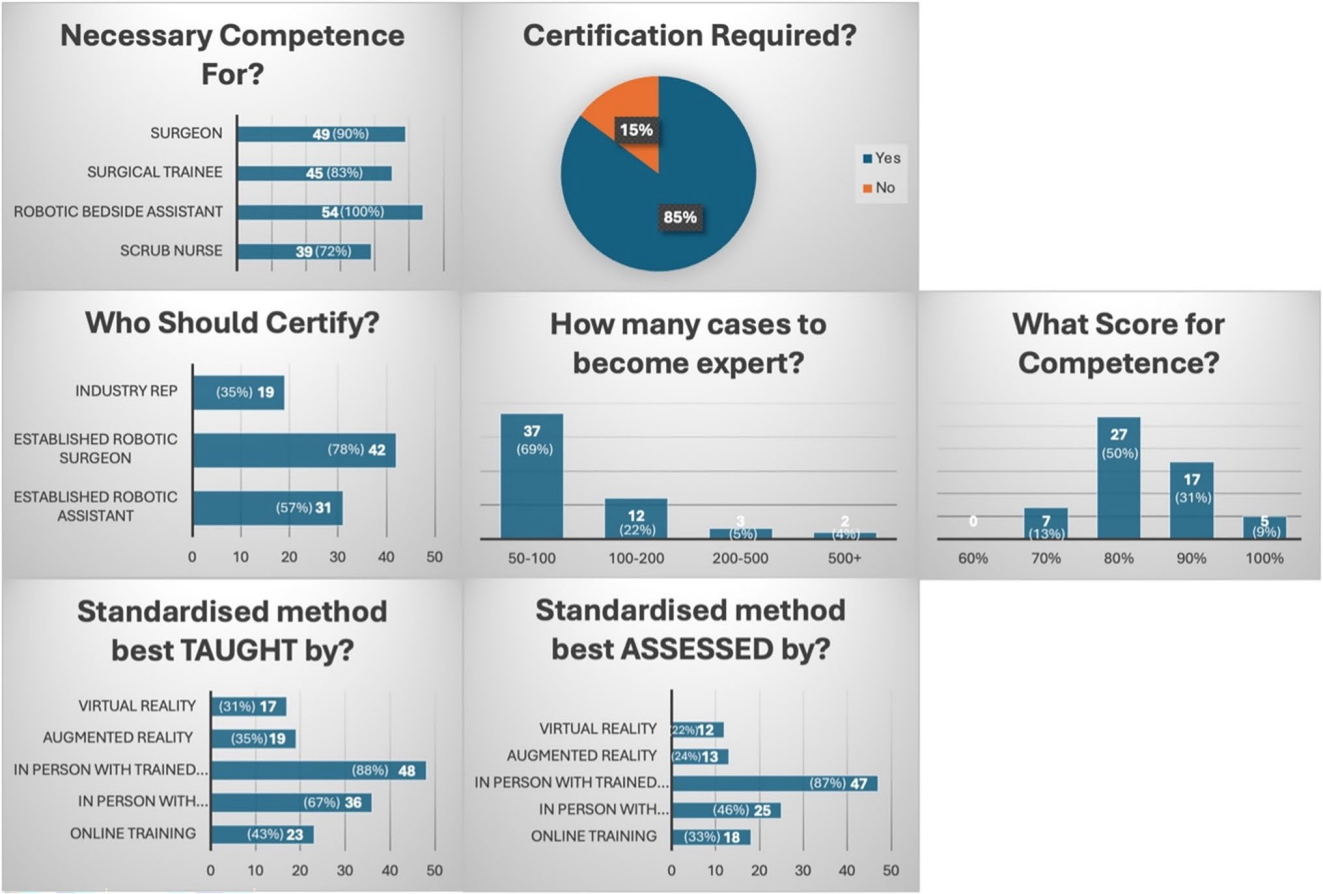
Graphical representation of accreditation survey results. Full descriptions for standardised methods taught/assessed by: In person with trained healthcare professional and in person with industry representative

Table 3 summarises the Delphi consensus. These 5 sections represent the agreed steps to safely and reliably set up the system. There was high (> 90%) agreement on the importance of adhering to core port placement principles (e.g. target anatomy and spacing of ports) and uniformly endorsed best practices for docking, instrument exchanges, and undocking procedures. Six statements did not reach the 80% consensus threshold and were excluded from the final protocol. These included advanced or infrequently used features (e.g. routine tracking of instrument lifespan on the vision cart and use of a 30° upward camera view for instrument insertion). However, agreement was obtained on fundamental knowledge and skills—for example, naming all robotic system components and controls, understanding emergency undocking procedures, optimising patient positioning, and ensuring safe removal of all instruments before undocking. All such agreed elements were incorporated into the final accreditation checklist (Fig. 1).

**Table 3:** Summary of accreditation protocol

Phase	Components
System knowledge	Identify all components of the robotic system (patient cart, vision cart, surgeon console) and explain the buttonology (e.g. port clutch, instrument clutch)
	Demonstrate knowledge of endoscope handling and settings—for example, correctly describe how to target the endoscope (camera) and adjust 30° scope orientation
	Outline the emergency undocking procedure (e.g. describe actions if an urgent conversion is required), including use of the emergency release key to open instrument jaws and the manual cart drive lever
	Confirm proper operating table setup and integration and optimise patient positioning to prevent collisions when docking
Port placement	Given a target anatomy, mark or identify appropriate port sites, including the endoscope port (approximately, 10–20 cm from target) and instrument ports (spaced ~ 6–8 cm apart) with consideration of bony landmarks and the assistant port distance (minimum 7 cm away from robotic ports)
Docking and driving in	Position the patient cart for docking: align the robot patient cart correctly with the patient and drive it into position using the guided laser or centre column alignment, maintaining appropriate clearance
	Dock the endoscope arm to the camera port first, attaching the endoscope and securing the port. Ensure the endoscope horizon is neutral and the camera cable is looped safely without tension
	Dock instrument arms sequentially to their respective ports. Dock each robotic arm without causing joint overextension or collision, adjusting boom height or arm spread if needed. Verify that each arm is appropriately spaced with no system error messages occur
Instruments and changes	Perform a safe instrument exchange: remove a robotic instrument from a designated arm and replace it with another as instructed. Ensure the instrument is straight and not articulating when removing, using reverse communication emphasising instrument name and arm number
	Demonstrate correct use of guided tool change feature (if available). Confirm control to the surgeon at the console only once the new instrument is in place and confirmed ready
Undocking & driving out	Safely remove all instruments and endoscope from the patient prior to undocking the robotic arms after reverse communication to confirm
	Undock each robotic arm from its port in a controlled manner and proceed to driving out when safe. Stow patient cart correctly

## Accreditation protocol for robotic setup

Based on the consensus results, an accreditation protocol was developed (Appendix 1). A narrative was written in conjunction with the steering committee to ensure standardisation in assessment methods. Each statement of the checklist represents one point, with a pass mark of 80% set based on survey results. Sections were not explicitly weighted; however, more significant sections such as port placement, driving in and docking, and instrument sand changes are weighted higher due to the higher number of statements. A free online platform (Google Forms) was employed to make an accessible user interface with real-time score calculation. A pre-requisite of equipment required is illustrated, with the form able to be utilised to train and assess robotic system setup.

## Discussion

At present, the majority of robotic surgery training emphasises the development of surgeon console skills. Industry-led training programmes often lack clinical context and adaptability. Conversely, surgeon-led courses provide valuable practical insights and experiential learning, although this may vary significantly due to teaching approaches. The consensus addresses these limitations by establishing a standardised, expert-endorsed curriculum specifically for the setup and docking of a robotic platform. We have identified the key steps required and formulated these into a usable format. This can be applied to not only the accreditation but also to guide and standardise training in robotic setup.

The Delphi consensus results corroborated the need for a standardised method of setup. The majority of respondents were consultants involved with high-volume practice. However, the respondents had a geographical bias towards Europe, with the largest proportion of responses originating from the UK, followed by Italy and Belgium. This may be a weakness with respect to external validity; however, it is unlikely setup practice varies significantly between continents. Furthermore, whilst the majority of respondents were consultant/attending level, a significant proportion were first assistants. This represents the individuals who are intimately involved with the setup and troubleshooting of the system. The views of these aforementioned experts were also sought as to accreditation in robotic setup.

A substantial proportion of respondents expressed strong support for mandatory certification, with a preference for this to be overseen by established robotic surgeons. Furthermore, the robotic bedside assistant role received the highest level of support for accreditation. In-person training and assessment, notably by trained healthcare professionals rather than industry representatives, was preferred, underscoring the perceived importance of direct clinical oversight, likely informed by the respondents’ extensive clinical experience. Most respondents identified competence thresholds of eighty to ninety percent on checklist-based evaluations as appropriate, suggesting a pragmatic balance between excellence and achievability.

System knowledge was heavily emphasised by the panel. High levels of agreement were achieved on the requirement for assistants to have knowledge of all system components and functions. Despite this, consensus was not reached on factors such as needing in-depth knowledge of the vision cart’s advanced functions (for example, managing instrument lives or audio features). This may be more relevant to the wider surgical team, rather than the bedside assistant. Consensus on core port placement principles and docking technique would minimise arm clashing and maximise range of motion. The accreditation protocol task of planning port placement can be applied as a verbal or simulation exercise during training, even if the final responsibility for port sites often lies with the surgeon. During docking, consensus statements underlined careful alignment of the patient cart, the importance of docking the endoscope first to obtain a stable view, and methodical attachment of the instrument arms with attention to avoiding collisions.

The instrument exchange and undocking phases similarly highlighted strong agreement on best practices. All participants agreed instrument changes must be performed with coordinated communication and attention to safety. This was achieved by ensuring the instrument is straight and off tissue when removing. Similarly, during the final phases of a procedure, verifying all instruments are removed, the endoscope light turned off to prevent fire risk, and that it is safe to move the robot away were endorsed by over 95% of experts. Incorporating these checks into an accreditation framework ensures that vigilance about patient and equipment safety.

Emergency protocols were highlighted as salient. There was strong agreement that assistants must understand how to handle system failures and emergent undocking situations. Interestingly, the respondents did not reach consensus on recommending that specific team roles be assigned in every team briefing. This may possibly be due to emergent conversions being rare. Whilst a granular definition of emergency undocking was beyond the scope of this, our unit’s quick reference protocol (Table 4).

**Table 4:** Quick reference emergency undocking protocol [13]

	Controlled Emergency Undock	Uncontrolled Emergency Undock
Situation	Bleeding controlled with manual pressure of robotic arm	Bleeding unable to be controlled robotically Cardiorespiratory compromise
Consider	Robot cannot be driven away if one arm remains docked Lateral arm (ideally ipsilateral side to patient cart) to be used for control of bleeding to allow space for surgical team	Prepare sterile tray for conversion Use two hands to remove Two instruments at one time Additional help
Roles and responsibilities	1. Surgeon to alert and scrub 2. Scrub nurse to prepare sterile tray for conversion 3. Circulator to drive robot away monitoring for clearance 4. First assistant to remove instruments and undock. Care to not remove instrument controlling bleeding	1 – 3: As-controlled undock *If first assistant and scrub nurse on ****same**** side:* First assistant to remove instruments and undock two arms on *opposite* side Scrub nurse to remove instruments and undock two arms on *same* side *If first assistant and scrub nurse on ****opposite**** sides:* First assistant and scrub nurse to remove 2 instruments and undock arms on respective sides
Troubleshooting	**Faults** *Recoverable* – Orange lights – Can press “recover fault” button on surgeon console touchpad, patient cart touchpad or vision cart screen *Non-recoverable* – Red lights – Power cycle to restore error. Not required to remove instruments / endoscope **Use of emergency key in case of instrument failure** Locate manual instrument release kit within sterile tray Insert Allen key into grip release section of instrument housing Turn in direction of arrow *Note instrument should not be reused after using the instrument release tool* **Use of manual drive if power loss** *NB: Ensure robot is not docked to patient* Open access panel on front of patient cart base Turn red lever to enable manual cart driving Two people to move cart due to weight (840 kg) Return red lever to original position (powered)	

The emphasis on using reverse communication (two-way verbal confirmations) throughout was highlighted. The non-technical skills aspect in robotic surgery is a pertinent one. Lack of direct vision, reliance on team members, and noise from equipment are just a few of the new factors involved with robotic surgery [10]. Therefore, effective communication is paramount. The concept of reverse communication to repeat back instructions was emphasised by the consensus at almost all stages. Driving in and docking, instrument insertions and exchanges, and driving out all employ an emphasis on reverse communication for accreditation. This practice enhances team situational awareness and reduces mistakes.

## Limitations

The Delphi methodology has inherent limitations. The initial set of statements was generated by a small steering committee, which introduces potential bias. We attempted to mitigate this by allowing participants to suggest additional items in Round 1; however, it is possible that some relevant tasks were not included or were framed in a way that influenced voting. Moreover, expert consensus represents a relatively low level of evidence; the protocol is based on collective opinion and experience rather than empirical outcome data. Additionally, the expert panel, whilst international, had a majority of participants from Europe (84%); practices in other regions or with different technology could vary. Estimates report over 60% of worldwide robotic systems are installed in the USA [11]. This may limit the external validity of the established consensus.

Furthermore, a checklist assessment with over 80 points could be time-consuming. In busy clinical environments, a balance must be struck between thorough evaluation and practical feasibility. Ongoing refinement and simplification of the protocol may be warranted, focussing on the most safety–critical steps for accreditation. Before implementing this accreditation framework widely, it should be validated—for example, by assessing whether individuals certified via this protocol indeed perform setup tasks more efficiently or with fewer errors in real cases.

Another limitation is that our consensus is platform specific. As noted, certain steps, especially related to port placement measurements and the physical act of docking, are particular to this system’s design. Although this may have applicability to future iterations of the da Vinci multiport system, it may not be translatable. With the surgical robotics field diversifying, a significant limitation is that this does not offer a platform-agnostic setup guide. However, owing to the specific technological and engineering aspects of each robotic platform, it may be that this methodology be adapted to other systems. Further work, with similar expert consensus, to identify platform-specific clinically oriented best practices may be warranted. Moreover, this protocol is not validated; a period of piloting is required prior to fulfilling the domains within Messick’s validity [12].

## Conclusion

An international consensus-based accreditation protocol for robotic surgical setup has been achieved. Through a Delphi process, experts identified best practices for robotic system preparation, docking, instrument handling, and undocking. The resulting protocol provides a structured checklist of tasks that can be used to train and assess bedside assistants or other team members responsible for setting up the robotic patient cart. Implementing such a protocol can help standardise training across institutions, thereby minimising variability and enhancing the safety and efficiency of robotic surgery programmes through accreditation. Future efforts should validate the protocol’s effectiveness in improving performance and explore its adaptation to additional robotic systems.

## Figures and Tables

**Fig. 2:**
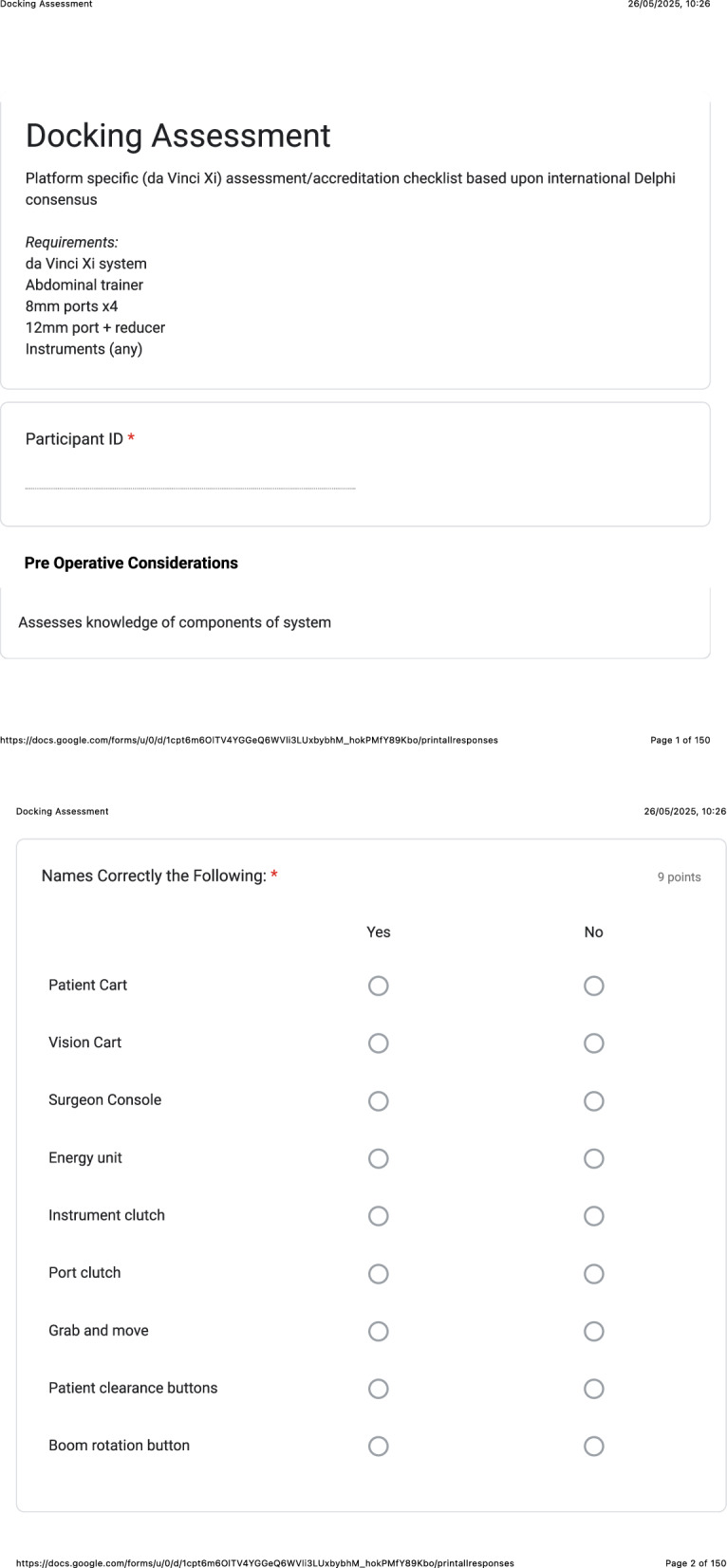

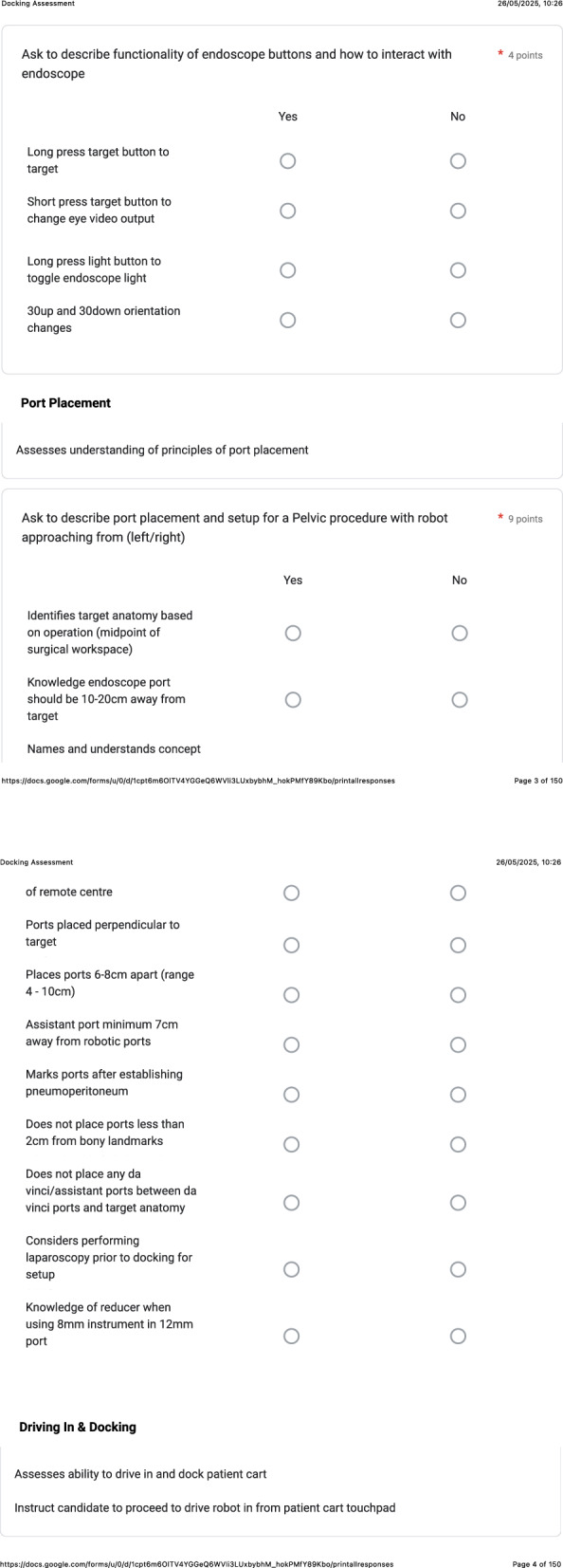

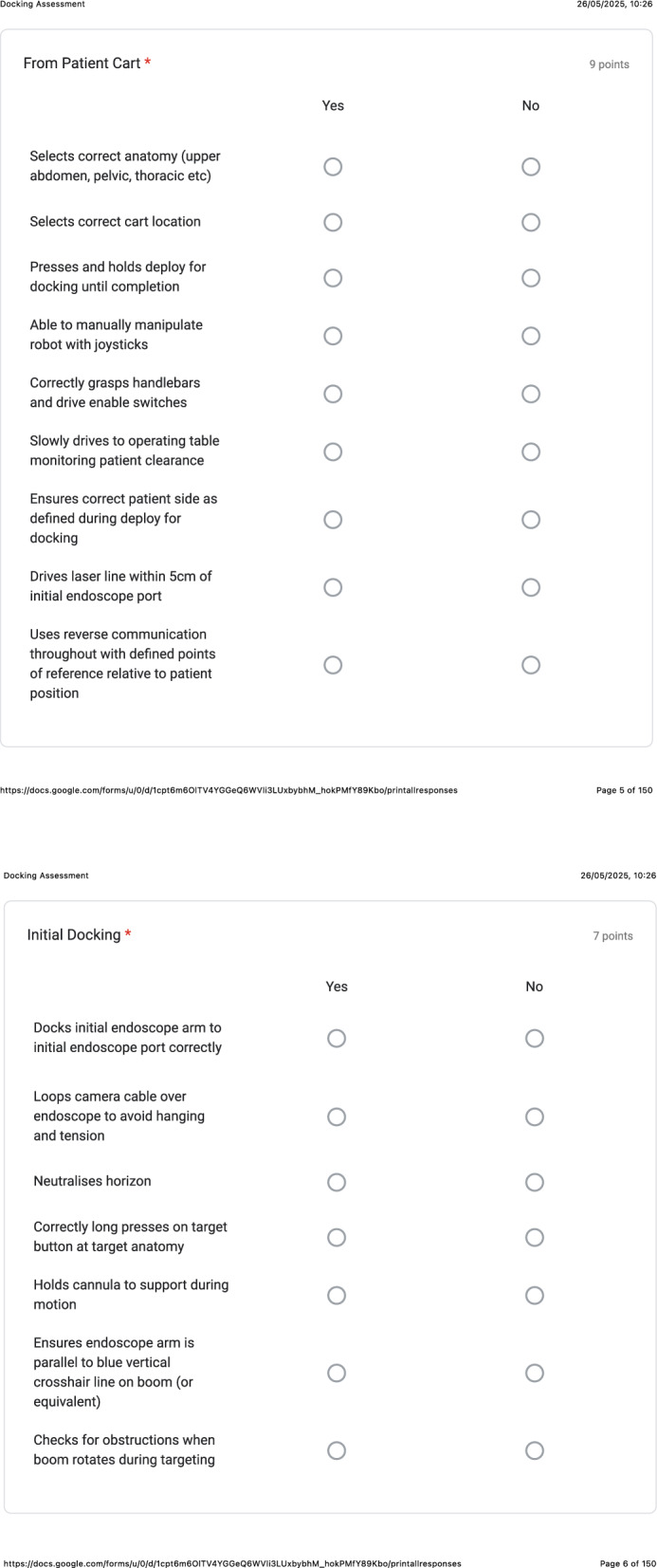

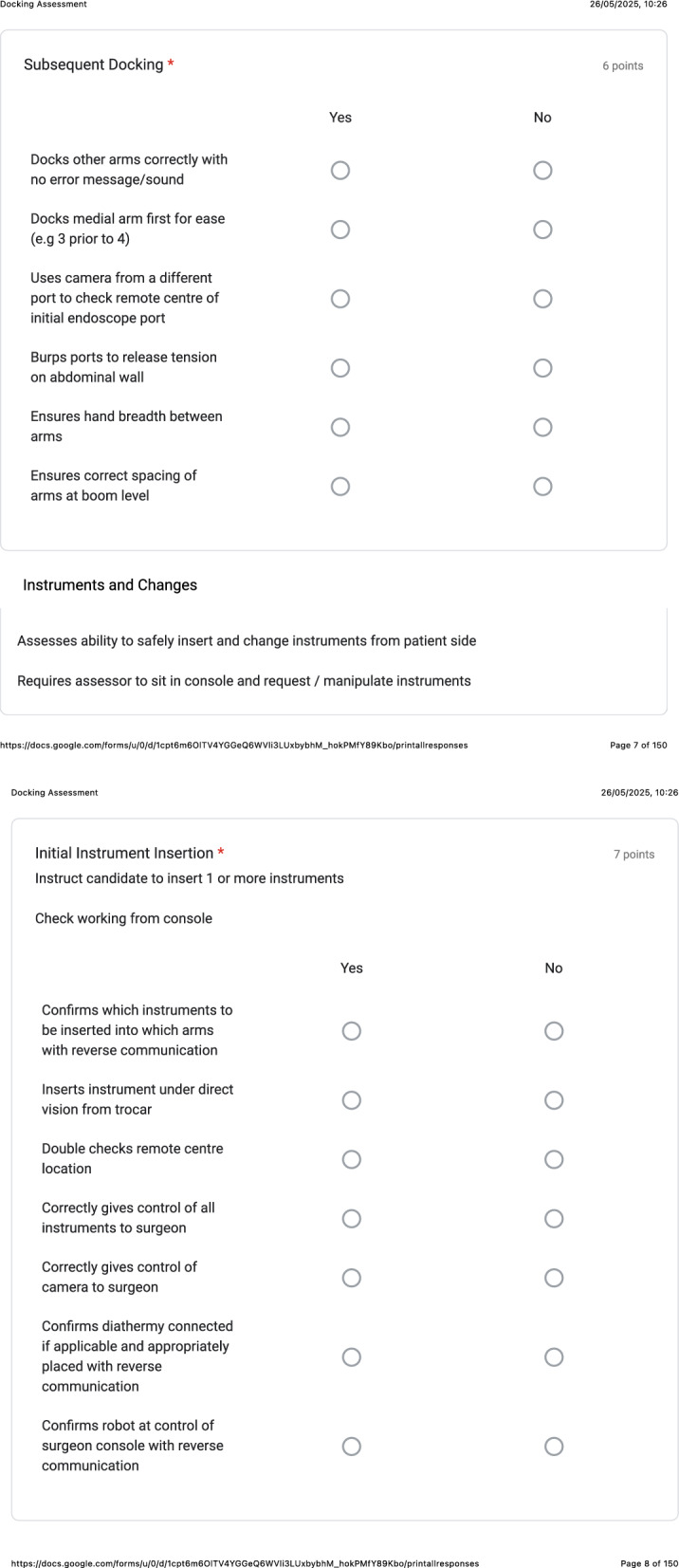

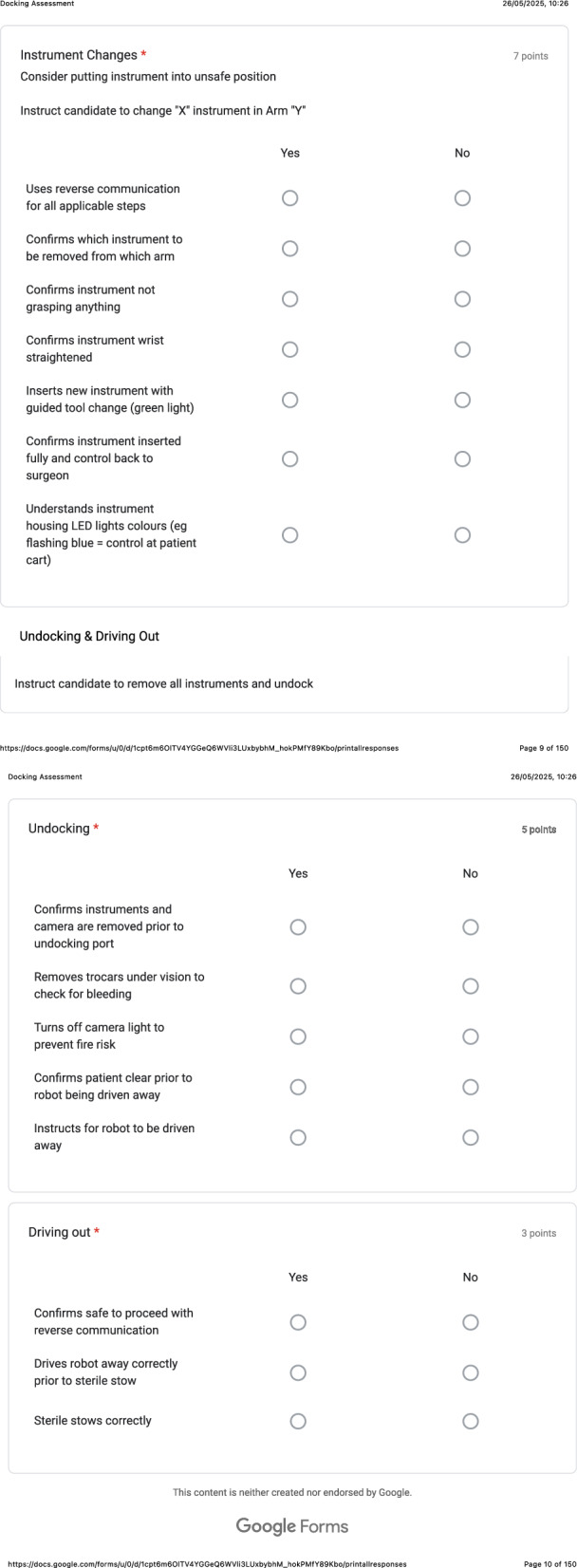
Accreditation protocol

## Appendix

See Fig. 2
